# Downstream changes in river avulsion style are related to channel morphology

**DOI:** 10.1038/s41467-020-15859-9

**Published:** 2020-04-30

**Authors:** J. M. Valenza, D. A. Edmonds, T. Hwang, S. Roy

**Affiliations:** 1 Department of Earth and Atmospheric Sciences, Indiana University, 1001 East 10th Street, Bloomington, IN 47405 USA; 2 Department of Geography, Indiana University, 701 E Kirkwood Avenue, Bloomington, IN 47405 USA; 3 Planet Labs, 645 Harrison Street, San Francisco, CA 94107 USA

**Keywords:** Hydrology, Natural hazards, Geomorphology

## Abstract

One of the most dramatic events in river environments is the natural diversion, or avulsion, of a channel across its floodplain. Though rarely witnessed, avulsions can cause massive floods, and over geologic time they create most of the fluvial stratigraphic record. Avulsions exhibit behavior ranging from reoccupying abandoned channels to constructing new channels and splay complexes. To quantify avulsion behavior, or style, we measure avulsion-related floodplain disturbance in modern environments. We show that for 63 avulsions from Andean, Himalayan, and New Guinean basins, avulsion style correlates with channel morphology and changes systematically downstream. Avulsions in braided rivers reoccupy abandoned channels, whereas avulsions in meandering rivers often produce flooding and sediment deposition during channel construction. These downstream changes in avulsion style can explain the abrupt transition from channel-dominated to floodplain-dominated facies commonly observed in foreland basin stratigraphy. These dynamics also explain why some avulsions are more hazardous than others.

## Abstract (web summary)

River avulsions are dramatic events that can cause the loss of many human lives. The authors here investigate how river avulsion style changes with river morphology, and how these changes impact flooding and stratigraphy.

## Introduction

Alluvial rivers usually migrate through shifting bars and eroding banks, causing channels to braid and meander. Occasionally, this incremental migration is interrupted by natural channel diversion, or avulsion^1–3^, which results in a complete channel relocation across the floodplain. Avulsions occur when sediment accumulates on the channel bed, elevating it above the surrounding floodplain^2,4^. In this superelevated position, channel banks and levees are prone to erosion by overbank flow events. Once banks and levees begin to erode, water escapes the channel, and under the right conditions^2^ this induces more levee erosion until all the flow is diverted. This diverted flow establishes an avulsion channel in one of two generally accepted styles, defined by the extent of floodplain disturbance^1,3–7^. Avulsions that direct flow into a pre-existing channel, resulting in little to no floodplain disturbance, are considered annexational. Incision may accompany annexation as the channel adjusts to a new flow regime, but otherwise the floodplain remains relatively undisturbed. Alternatively, avulsions that construct a new channel through phases of sediment deposition and reworking are considered progradational.

Understanding the environmental factors that control avulsion style is important for predicting channel migration as well as the characteristics and distribution of floodplain sediments. However, avulsions occur infrequently^8^ and often in remote locations. Because of this, avulsions are rarely directly observed, which has made it difficult to identify the factors that determine avulsion style. Identifying these factors would enhance our interpretation of fluvial avulsion deposits in the geologic record and help mitigate avulsion-related flooding hazards to communities built on floodplains.

To address these challenges, we developed a new remote-sensing method called fingerprinting to quantify how avulsions disturb the landscape. We construct avulsion fingerprints from yearly composited Landsat data, which is then classified with a Tasseled Cap transformation, and filtered to isolate areas disturbed by the avulsion (see methods for more detail). With an avulsion fingerprint we can measure the extent of floodplain disturbance and quantitatively define the avulsion style. We calculate fingerprints of 63 avulsions across three sedimentary basins with drastically different climates and geologic settings. Avulsions in all three basins behave similarly: multithread, braided channels near mountain fronts have small, compact fingerprints, while single-threaded, meandering channels in distal settings have larger, extensive fingerprints.

## Results

### Avulsion site selection and fingerprinting

**Fig. 1:**
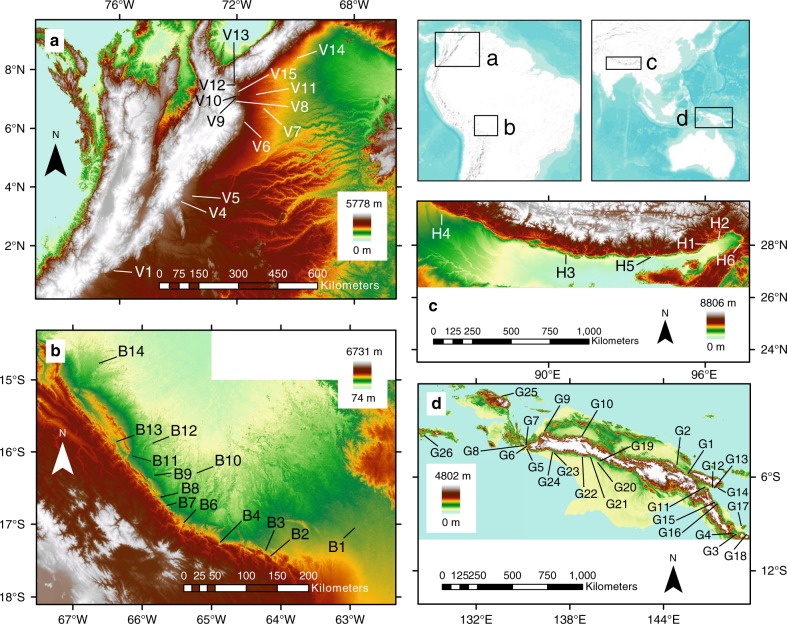
Avulsion locations across three basins. **a** Northern Andes foreland, including Colombia and Venezuela; **b** Central Andean foreland in Bolivia (an additional sample not shown here, B15, is located on the Argentine-Bolivian border to the south of map); **c** Himalayan foreland, India and Nepal; **d** New Guinean basins in and around the Central Highlands. Locator map in upper right shows locations of studied basins. Bare-Earth SRTM DEM is from Jarvis et al.^70^.

To isolate fluvial processes from delta and shoreline processes, we focus on events occurring upstream of delta environments. We selected 63 avulsion events where all or most of the avulsion activity is captured by Landsat imagery from 1986 to 2017 (Fig. 1). We identify avulsions in meandering reaches as the diversion of flow from a parent channel and the establishment of a new channel outside of the active meander belt. Braided reaches require more precise criteria, as braid threads often exhibit rapid change and migration without avulsion. To consider an event avulsive, we look for the appearance of new channel threads outside of the active bankfull braid belt, and also the abandonment of parent threads. This definition excludes the temporary increase of the number of braid threads associated with stage-dependent flooding behavior^9,10^.

Of our 63 avulsion events, 45 events are newly described while 18 are described by refs. ^11,12^. There are 30 avulsion events within the greater Andes foreland, 14 of which occur within the Beni and Chaco plains of the Central Andean foreland basin (Bolivia and Argentina)^13^ and 16 of which occur within the Llanos and Barinas-Apure basins (Colombia-Venezuela)^14^. Within the Himalayan foreland basin, we selected four events in northeastern India, along with two events in Nepal. We selected 27 events in the various New Guinean basins within the complex convergence zone between the greater Australian and Pacific plates^15^. All study areas experience distinct wet and dry seasons in humid tropical to subtropical climates^16–19^.

**Fig. 2:**
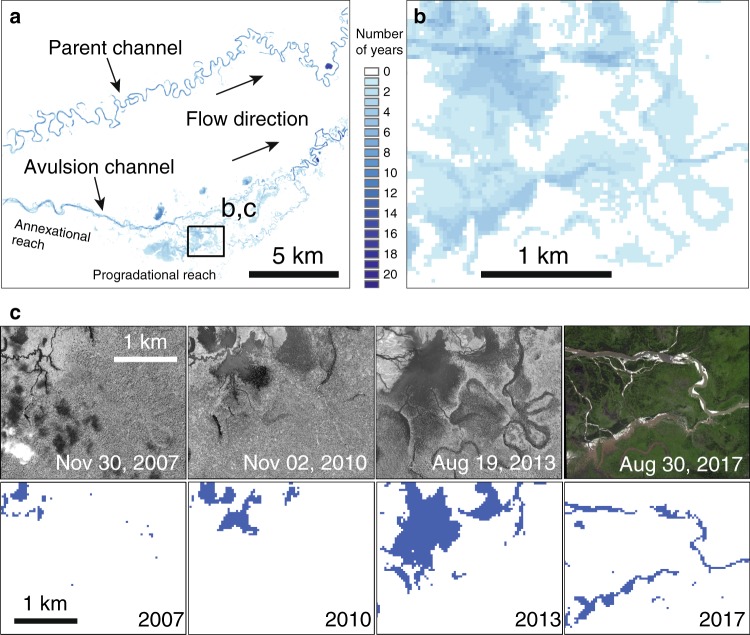
Qualitative validation of the avulsion fingerprinting process. **a** Avulsion fingerprint map of the central part of avulsion B12 (Moleto River, Bolivia). Inset square shows area used in the validation process. Number of years corresponds to the number of times a given pixel is included in the avulsion fingerprint. **b** Close-up of the avulsion fingerprint map over the area of interest used for validation. **c** Four years showing high-resolution satellite imagery (upper row) and the corresponding single year avulsion fingerprints (lower row). Dark blue pixels represent detected avulsion pixels. The four successive fingerprints record the progradation of the crevasse splay associated with the avulsion. Note that more than just these fingerprints were used to calculate the fingerprint in **b**. Satellite images in **c** are World View 1 panchromatic images and World View 1 RGB images. Imagery Copyright 2017 DigitalGlobe, Inc.

To define the style of these 63 events we use an avulsion fingerprinting routine that is based on two premises. The first premise is that avulsion activity will temporarily disturb land cover. Indeed, we see recognizable spectral shifts during avulsion events in remote-sensing data (Supplementary Fig. 1). Such shifts indicate flooding, vegetation burial and die-off, and exposure of soil through vegetation reduction or sedimentation. While these are generally good avulsion indicators, not all avulsion activity is observable as a spectral shift. For instance, avulsion-related flooding or sedimentation could go undetected if it occurs under forest canopy without altering vegetation cover. Thus, our avulsion fingerprints represent a minimum estimate of avulsion activity. The second premise is that the avulsion fingerprint is a reasonable proxy for sediment deposition. This should be true because the fingerprint depends on the flooded area, which is the area that could receive sediment (see methods). Indeed, when we compare the avulsion fingerprint to high-resolution images where sediment deposition is visible (Fig. 2 and Supplementary Fig. 2), we see that the fingerprints identify areas of deposition.

### Downstream changes in avulsion style

Avulsion fingerprints allow us to measure the extent of floodplain disturbance and quantitatively define avulsion style as a ratio between the area of the parent channel and that of the avulsion fingerprint. We measure four variables associated with each avulsion event. The first variable is a quantitative avulsion style ratio (*S*_*R*_). To quantitatively define style, we compare the total area of avulsion activity from the fingerprint with the surface area of the active parent channel before the avulsion. This parent channel area is measured over the distance between the point of avulsion initiation, or avulsion site, to where it rejoins the parent channel downstream, or in cases where the avulsion channel does not rejoin the parent channel, the distance traveled by the new avulsion channel (see Methods section for more detail). In terms of traditional descriptors for avulsion style, *S*_*R*_ has a value close to 1 for annexational or incisional avulsions that reoccupy pre-existing channels and/or incise into the floodplain, and a value much greater than 1 for progradational avulsions that disturb the surrounding floodplain. The second variable is the distance from the avulsion site to the mountain front following the channel-belt centerline. We define the mountain front as the physiographic transition between high-relief, bedrock-dominated mountainous catchments and low-relief, unconfined plains dominated by sediment accumulation. To compare rivers of different sizes we normalize these distances by the average channel width at the avulsion site. Without discharge data, channel width is the most useful measure of river size available through remote sensing. The third variable is the slope of the parent channel-belt centerline near the avulsion site. We measure the channel-belt centerline slope over the smallest distance that produces a recognizable slope (typically 5–10 km but may be longer for systems on very low slopes) using SRTM elevation data. The fourth variable is the number of flow threads within the channel belt at the avulsion site. We measure this on the parent channel prior to the avulsion at multiple locations around the avulsion site and use it to classify each river as braided (multiple threads), meandering (single thread), or transitional (alternating reaches with single and multiple threads). Fingerprint data used in this study are available in Supplementary Data 1.

**Fig. 3:**
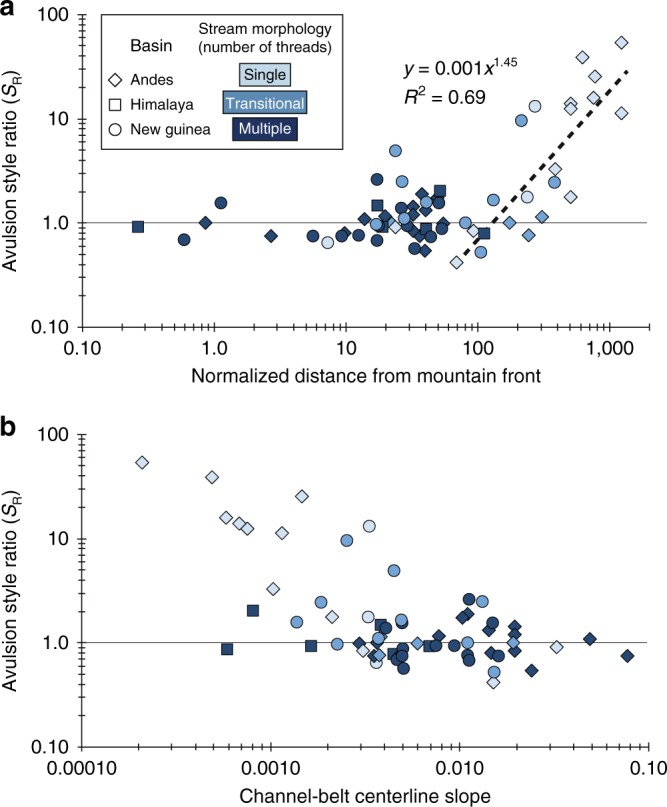
Avulsion style depends on downstream distance and slope. **a** Avulsion style ratio (*S*_R_) is positively correlated with the normalized distance from the mountain front (distance/channel width). For distances < ~60, *S*_R_ ranges between ~ 0.5 and 2.5, except for one outlier with *S*_R_ 4.9. These low *S*_R_ values indicate annexation-dominated avulsion. Beyond a normalized distance of ~60–100, *S*_R_ exhibits a strong positive correlation with distance. These higher *S*_R_ values indicate avulsions with significant progradation. Only samples with *x*-axis values >60 were used to calculate the best fit line. **b** *S*_R_ is negatively correlated to river slope. On slopes steeper than ~0.005, *S*_R_ is small and ranges from ~0.5 to 2.5. On slopes shallower than 0.005, *S*_R_ increases as slope decreases.

**Fig. 4:**
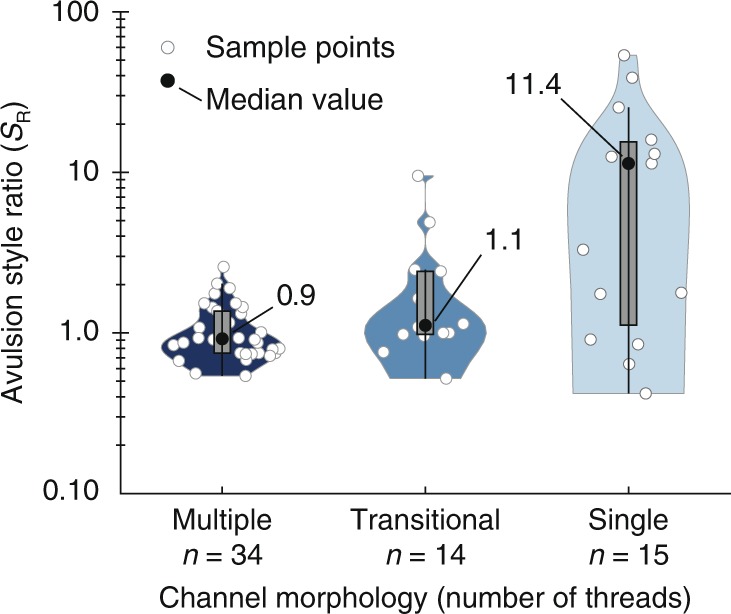
Avulsion style is related to channel morphology. Rivers with multiple flow threads have the smallest median *S*_R_ values. Avulsions in transitional channels, or those with alternating reaches of multiple threads and single threads, also have a small median *S*_R_, but they have a larger range than events in channels with multiple threads. Avulsions on single-thread channels have the highest median *S*_R_. Numbers refer to median values of the distributions, boxes show the 2nd to 3rd quartile ranges, and lines extending from boxes represent the 1st to 2nd and 3rd to 4th quartile ranges.

We find that avulsions in all three basins behave the same way: avulsions that occur closer to mountain fronts generate small, compact fingerprints, while avulsions occurring relatively farther downstream produce increasingly larger fingerprints (Fig. 3a). For all avulsions in the study, *S*_*R*_ ranges from 0.4 to 42.4. *S*_*R*_ is nearly constant near the mountain front, varying mostly from 0.5 to 2.5. For the 22 events occurring beyond a normalized distance of ~60–100, there is a well-defined increase in *S*_*R*_ up to 42.4 (Fig. 3a). This trend of a generally constant *S*_*R*_ followed by an increase beyond a normalized distance of ~60–100 is remarkable insofar as it holds across three different basins without controlling for climate, geology, or tectonics. In addition, *S*_*R*_ correlates to channel-belt centerline slopes. Generally, *S*_*R*_ decreases with increasing slope, until slopes exceed 0.005, after which *S*_*R*_ generally stays below 2.5 (Fig. 3b). *S*_*R*_ exhibits differences among channel morphologies, and on average is highest for single-thread and lowest for multithread rivers (Fig. 4).

Our fingerprints show that there are avulsion events with exclusively annexational behavior (those with *S*_*R*_ ~1), but there are almost no events with exclusively progradational behavior. Annexation seems to be an important part of every avulsion in our dataset. Consider that even events with large *S*_*R*_, which we might call progradational, show localized channelization with no adjacent floodplain disturbance, a clear sign of annexation. For example, on a single avulsion on the Moleto River, Bolivia (sample B9) there are clear zones of both annexation and progradation (Fig. 2a). Near the avulsion site the fingerprint is confined to the pre-existing channel annexed by the avulsion. Farther downstream, the fingerprint becomes more extensive, as progradation causes widespread land-cover disturbance. Within this disturbance, we observe finely detailed distributary networks created by crevasse splays, which are common features of progradation (Fig. 2b). This illustrates the variability of avulsion style within an event that would be conventionally labeled progradational.

## Discussion

The change in avulsion style we observe in our data is coincident with the change in channel morphology from braided to meandering^20–22^ (Fig. 4, Supplementary Fig. 3). We suggest that the downstream changes in slope and grain size that shift channel morphology from braided to meandering also cause the shift in avulsion style we observe in Fig. 3. In the absence of tectonic complexity, rivers generally shift from multithreaded, braided streams in steeper-sloped upstream locations to single threaded, meandering streams at lower slopes farther into the basin^23–25^. This shift occurs because when channels leave the mountainous catchment and encounter the shallower slope of the sedimentary basin, their stream power^26,27^ and competency^28,29^ also decrease. This leads to the selective deposition of coarser grains^20,29^, which creates a channel and floodplain substrate with low-cohesion, resulting in a braided channel. Farther downstream, as selective deposition of coarser grains continues, the sediment load effectively runs out of gravel and the proportion of fine-grained sediment increases^30–32^. This eventually results in floodplains with more cohesive sediment, and channels that are narrower and deeper. Narrower and deeper channels stifle bar development, and promote overbank floodplain deposition^33,34^. The net effect is to reduce chute channel formation across bars, maintain channel sinuosity, and shift the channel from braided to meandering^21,32,35^.

Low *S*_*R*_ avulsions occur in braided reaches because abandoned channels persist as attractive pathways for future avulsions due to the lack of fine-grained overbank sedimentation that would otherwise fill this accommodation space. Furthermore, the braided rivers in our study have steeper slopes (Fig. 3a and Supplementary Fig. 3), which should promote more rapid channelization of overbank flows^36^. Additionally, if the braided river floodplains have non-cohesive substrates, then it is possible some of these avulsions are not driven by annexation, but by incision^6^_._ This could occur because a new channel may rapidly incise into the easily erodible, non-cohesive substrate, producing a compact fingerprint similar to an annexational event. However, almost all low *S*_*R*_ events exhibit channelized flow patterns from initiation through completion. This suggests that even in potentially incisional cases the stream path is determined by pre-existing channels.

Higher *S*_*R*_ avulsions occur on meandering rivers because there is more fine-grained material in the river sediment load and there are lower slopes. Floods distribute fine-grained material across the floodplain, preferentially filling the accommodation space in abandoned channels. This has a smoothing effect that may fill some abandoned channels, reducing their potential to become avulsion pathways. This effect probably does not eliminate all potential pathways, as recent work shows floodplains have substantial negative relief^37–39^. However, even partial smoothing could decrease connectivity of potential pathways on the floodplain and force progradation where channels cannot fully accommodate avulsion flow. Perhaps more importantly, fine-grained sediment and vegetation increase riverbank strength and stabilize meandering rivers^21,40,41^. This promotes vertical aggradation, or superelevation, at the expense of lateral migration of the river^42^. When an avulsion finally does occur, the superelevated river discharges most of its water and sediment onto the floodplain. Because fine-grained sediment is more easily transported, it travels farther across the floodplain before settling out and becoming available for bar and channel construction. In these conditions, avulsions begin as crevasse splays and develop into prograding distributary channel networks. This continues until the prograding distributary network encounters a channel that can accommodate diverted flow. If a floodplain experiences depositional smoothing, then the distributary network will prograde farther before encountering an accommodating channel, creating a larger fingerprint.

**Fig. 5:**
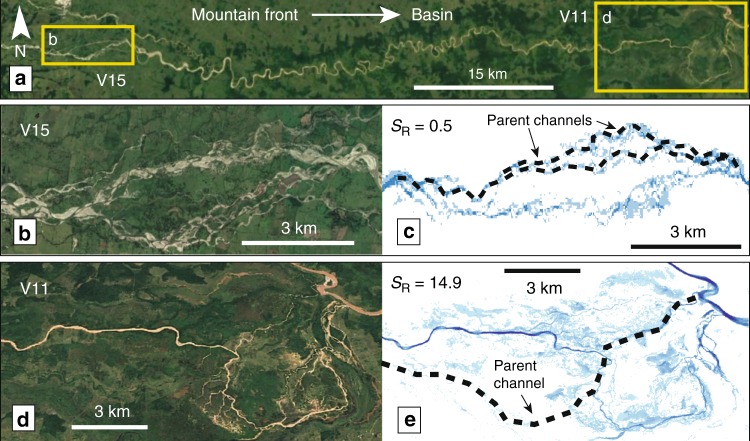
Two avulsions on the Sarare River, Venezuela with different styles. **a** Avulsion sample V15 occurs close to the Andean range front, exhibits a braided, multithread channel morphology (**b**), and has a compact fingerprint (**c**). Avulsion V11 occurs in a more distal location, exhibits a meandering, single-thread channel morphology (**d**), and has a larger fingerprint (**e**). Black dashed lines in **c** and **e** mark the parent channel location prior to avulsion. In all maps flow is to the right. Satellite imagery from Google Earth Pro 2019.

Samples V11 and V15, which occur on the same river, illustrate the relationship between channel morphology and avulsion style. In this case, V15 has an *S*_*R*_ = 0.5 in the upstream braided reach, while farther downstream in the meandering reach V11 has an *S*_*R*_ = 16.0 (Fig. 5). This shows that two avulsions ~60 km apart on the same river can have drastically different styles. Moving downstream, bed and suspended load grain sizes can change rapidly^43^, which could lead to the expression of different channel morphologies and avulsion styles in the same river.

An important implication of downstream changes in avulsion style is the potential to drive stratigraphic changes in foreland basins. Most of the avulsions identified in this study occur in distributive fluvial systems (DFS), which have been proposed as predominant contributors to foreland basin stratigraphy, especially in proximal areas^44,45^. Alluvial stratigraphy in foreland basins often displays a conspicuous stratigraphic upward shift from floodplain-dominated muddy facies to channel-dominated sandy facies^46–49^. This shift in stratigraphy has been attributed to changes in climate, floodplain aggradation rate, vegetation, thrust front migration, and sediment and water supply^30,50–56^. However, recent analysis of modern DFS describe a downstream shift from braided to meandering channel morphology^23–25,45,57^. If our remote-sensing results apply to the stratigraphic record, then they provide an autogenic mechanism for creating these different facies and suggest that there should be a predictable downstream shift in sedimentary facies on a DFS because avulsion style changes. Proximal avulsions annex pre-existing channels and would produce amalgamated channel-dominated facies. Distal avulsions create prograding distributary networks and would produce isolated channels surrounded by crevasse splay and heterogeneous overbank facies within the floodplain facies. This model does not require allogenic forcing, because under steady boundary conditions of water and sediment input, a DFS would aggrade and initially fill accommodation space, and then begin prograding into the basin. This would stack proximal, annexation-dominated braid plains on top of distal, progradation-prone floodplains^24,25,49^ and explain the facies shift without changing climate, aggradation rate, vegetation, thrust front migration, or sediment and water supply.

In the stratigraphic record this facies shift is abrupt^28,46,53^, which is also something we capture in our *S*_*R*_ data. In New Guinean and Andean basins, beyond a normalized distance of ~60–100 from the mountain front, *S*_*R*_ becomes significantly greater than one and grows larger with increasing distance (Fig. 3A). Exactly why *S*_*R*_ changes at this distance and increases in this fashion is not yet clear, though we hypothesize that this position and abrupt shift may be related to a river’s coarse-grained transport limit, known as the gravel-sand transition^58^. This transition is associated with rapid wash load fining^43^, braided to meandering channel transitions^58^, and topographic shifts from steeper to lower slopes^59^. All else being equal, downstream of the gravel-sand transition, lower slopes and finer-grained suspended sediment should promote high *S*_*R*_ avulsions.

Admittedly our timescale for observing avulsion events is short, which adds uncertainty when applying these results to the stratigraphic record. Ideally, we would want to know if our remote-sensing analysis captures the character of avulsions typically preserved in the stratigraphic record of foreland basins. Avulsions could be characterized by their recurrence interval or size. Unfortunately, we do not know the recurrence interval of these events, only that they have occurred recently. If avulsion area positively correlates to recurrence interval, then our data capture both frequent and rare events. By area, our avulsion fingerprints span five orders of magnitude from 0.1 km^2^ to 1000 km^2^. The largest avulsions have a channel-perpendicular span of ~10–20 km, and should be significant contributors to the stratigraphic record. Beyond stratigraphic architecture, these insights into the avulsion process also may inform hazard mitigation in modern settings. Avulsions are among the deadliest river-related hazards, and our data suggest avulsion-related flooding on single-thread, meandering rivers should be more severe. This is generally consistent with known avulsion events on the Indus River in 2010 (ref. ^60^) and the Yellow River in 1883 (refs. ^1^^,61^), which occurred in downstream reaches of the rivers and displaced millions of people. Still, avulsion hazard risk is not so simple—the Kosi River avulsion of 2008 occurred in a braided reach and still displaced millions of people^62,63^. Ultimately, the impact of a river avulsion will depend on the style of the event and the population in its path.

## Methods

### Avulsion fingerprinting methods

We developed a four-step fingerprinting process to measure the extent of land surface disturbance associated with each avulsion. The first step in the fingerprinting process is to collect all atmosphere corrected Landsat surface reflectance scenes (Level-2 data) that cover a given avulsion. Using Google Earth Engine (GEE), we select Landsat scenes covering the sample areas of interest from 1986 to 2017 (missions 5, 7, and 8), which provide multispectral images at 30-m spatial resolution. For all available scenes, we apply the GEE simple cloud score function, and mask all pixels that score above 20% probability of representing cloud cover. In cases where all pixels of a scene are above 20%, we remove that scene from that year’s scene collection. For each year, we then radiometrically align all available scenes and apply a median reducer that creates a single, yearly composite image. The median reducer reduces noise from seasonal or episodic spectral variations in vegetation, flow volumes, solar geometry, and atmospheric conditions, such as haze or remnant cloud cover. The median is applied to each pixel for all images during that year. By taking the median band value for each Landsat pixel, we create an image that represents the median surface conditions for that year. We complete this process for every year at every avulsion site in our data. Despite filtering for cloud cover and applying a median reducer, some yearly composites are noisy or contain incomplete spatial coverage. Noise at this step results mainly from remnant cloud cover and Scan Line Corrector errors (present in images from Landsat 7 after 31 May 2003), or incomplete scene coverage. These noisy yearly composites are removed from the stack.

In the second step, we transform each yearly Landsat composite into physically interpretable data by applying a Tasseled-cap transformation (TCT)^64–69^. Through this transformation we compress multispectral Landsat data into three principle components by applying preset coefficients calibrated for Landsat sensor type (TM, ETM+, and OLI, Supplementary Table 1). Transforming the data yields three principal components that represent physical surface properties, including brightness (TCB), greenness (TCG), and wetness (TCW). TCB represents the total reflectance by weight sum of all six Landsat bands (except for the thermal band), which emphasizes the brightest surfaces, such as bare rock or sediment. TCG was designed to capture the contrast between visible and near infrared (NIR) bands, which emphasizes the red edge spectral feature of green surfaces, such as healthy vegetation. TCW characterizes the contrast between the visible/NIR and short-wave infrared bands, which captures water-related spectral signals including surface water and soil moisture.

In the third step, we use TCT yearly composite images to identify pixels associated with avulsion activity. Our observations of avulsions suggest they affect the landscape by creating flooding (high TCW value), by killing off vegetation due to flooding inundation and channel relocation (low TCG value), or by increasing brightness because of sediment deposition or exposure of soil surfaces due to vegetation die-off (high TCB value). We use this commonly observed activity to define two different spectral signatures of avulsion-related pixels: high TCB and low TCG, or high TCW and low TCG. We isolate avulsion-related pixels by filtering yearly composites with TCB, TCG, and TCW value thresholds. Because each avulsion site displays variation in the spectral range, our thresholds are designed to capture extreme values in that scene. Avulsion-related pixels are defined as those having either: (1) TCB values above the 90th percentile and TCG values below the 50th percentile; or (2) TCW values above the 75th percentile and TCG values below the 50th percentile. These filters are calculated for each annual composite TCT scene. We then apply the filters to each yearly TCT image creating a set of binarized images where avulsion pixels detected with the TCB, TCW, and TCG thresholds have a value of 1, and non-avulsion pixels are 0. In addition to avulsion activity, these thresholds also highlight channels, marshes, and ponds. We use the identification of channels to create fingerprints of parent channels, which we use in conjunction with avulsion fingerprints to calculate *S*_*R*_. Marshes and ponds are excluded from fingerprints in a later step.

In the fourth step, we take each yearly TCT composite with avulsion-related pixels identified in step three and do a second composite by summing the image stack and creating the final avulsion fingerprint. Thus, locations with sustained avulsion-activity have higher values in the fingerprint (Fig. 2 and Supplementary Fig. 2). Any pixel with a value of one or greater is included in the final fingerprint. The final avulsion fingerprint only uses the yearly composites ranging from initiation to completion of the avulsion. Avulsion initiation is defined as the first yearly composite that exhibits flow diversion from the parent channel. We then identify the yearly composite that marks the end, or completion of the avulsion. This is defined as the first yearly composite that shows the parent channel completely abandoned, and the avulsion channel established as the new flow path. In some cases, these conditions are only partially met, where additional channel threads maintain a course through the vicinity of the parent channel, tributaries keep the parent channel from drying up completely, or establishment of the avulsion channel is still ongoing kilometers downstream from the avulsion initiation site. Avulsion duration varies widely and can take place in the span of 1 year, or it may take place over decades.

All information used in creating these fingerprints is in Supplementary Data 1.

### Calculating the avulsion style ratio

Our avulsion style ratio compares the area of the avulsion fingerprint to the area of what is essentially a parent channel fingerprint. The parent channel fingerprint is created using the same methods as the avulsion fingerprint. As a rule we used the shortest time ranges necessary to image the parent channel and the avulsion event, from initiation through establishment as the new primary flow path. Where possible, we used the same time ranges for avulsion and parent channel fingerprints, to avoid oversampling any lateral channel migration (meandering and braiding) not due to avulsion activity.

To calculate the avulsion style ratio, we define two masks, one around the parent channel, and another around the avulsion channel. Each mask is drawn manually to only include pixels clearly associated with the parent or avulsion channel, such as associated overbank activity in the avulsion fingerprint (for example, we exclude ponding, flooding, and channels that do not appear to connect with the avulsion channel(s)). When the association is not clear we consult the yearly image stacks to judge if certain portions are part of the avulsion. The masks cover both channels from the avulsion initiation site to the point where flow either rejoins the parent channel or joins a larger river system downstream. Because we know that activity associated with the parent or avulsion channel will generally cluster around those channels, we assume any pixels outside of the mask are likely false positives.. Additionally, pre-existing ponds, marshes, and poorly vegetated areas are highlighted by this workflow and these can occur near the channel. We mask these out if they are persistent and pre-existing bright or wet locations, because they would have existed before the avulsion.

The final avulsion style ratio is calculated by determining the number of all pixels within each mask of the parent and avulsion fingerprint with a value of 1 or greater. The avulsion style is given as the ratio of these two pixel counts (avulsion/parent channel). As mentioned in step 3 of the Avulsion Fingerprinting Method section, there are two spectral signatures we associate with avulsion activity; (1) TCB values above the 90th percentile and TCG values below the 50th percentile; or (2) TCW values above the 75th percentile and TCG values below the 50th percentile. We select the spectral signature that creates the smallest fingerprint. Thus, the associated avulsion style ratio is a minimum estimate of landscape disturbance by the avulsion. Avulsion style ratios are reported in Supplementary Data 1.

## Supplementary information

Supplementary Information

Description of Additional Supplementary Files

Supplementary Dataset 1

## Data Availability

All data from this paper, including median-reduced, Tasseled-cap-transformed, multi-year geoTIFFs, and parent and avulsion channel masks are available in a public repository (scholarworks.iu.edu) located at http://hdl.handle.net/2022/25284.

## Code availability

Code associated with this work is available in a public repository (scholarworks.iu.edu) located at http://hdl.handle.net/2022/25284.
