# Supplementary material for: Downstream changes in river avulsion style are related to channel morphology
Source: Nat Commun. 2020 Apr 30;11:2116. doi: 10.1038/s41467-020-15859-9 (PMC7192919; doi:10.1038/s41467-020-15859-9)
Supplement: Supplementary file 1 — Supplementary Information [file 41467_2020_15859_MOESM1_ESM.pdf]

# Downstream changes in river avulsion style are related to channel morphology

Valenza et al.

**<u>Contents</u>**

**Supplementary Table 1: Avulsion Style data**

**Supplementary Table 2: Tasseled Cap Coefficients**

**Supplementary Figure 1. Progression of land surface changes during progradational event**

**Supplementary Figure 2. Qualitative validation of results from avulsion sample B11, Bolivia**

**Supplementary Figure 3. Channel morphology is related to slope and normalized distance**

# SUPPLEMENTARY MATERIAL

| Avulsion ID | Lat | Lon | type | River distance (km) | Channel width (m) | Normalized distance | Channel-belt slope | Avulsion style ratio ($S_R$) | Link to avulsion location in Google Earth Engine |
|---|---|---|---|---|---|---|---|---|---|
| V1 | 1.10920 | -76.23480 | 1 | 7.1 | 198 | 35.9 | 0.00353 | 1.0 | https://earthengine.google.org/#timelapse/v=1.1092,-76.2348,11.4,latLng |
| V4 | 3.50400 | -73.87590 | 1 | 16.1 | 432 | 37.3 | 0.01100 | 1.9 | https://earthengine.google.org/#timelapse/v=3.504,-73.8759,11.4,latLng |
| V5_1 | 3.74260 | -73.54850 | 2 | 35.4 | 147 | 240.8 | 0.00375 | 0.6 | https://earthengine.google.org/#timelapse/v=3.7426,-73.5485,11.4,latLng |
| V5_2 | 3.73056 | -73.49434 | 2 | 41.9 | 138 | 303.6 | 0.00381 | 1.1 | https://earthengine.google.org/#timelapse/v=3.730559,-73.494341,11.4,latLng |
| V6 | 6.19340 | -71.77800 | 1 | 29.2 | 595 | 49.1 | 0.01028 | 1.8 | https://earthengine.google.org/#timelapse/v=6.1934,-71.778,11.4,latLng |
| V7 | 6.73000 | -71.25650 | 3 | 69.9 | 57 | 1226.3 | 0.00115 | 11.4 | https://earthengine.google.org/#timelapse/v=6.73,-71.2565,11.4,latLng |
| V8 | 6.94976 | -71.98327 | 1 | 13.9 | 349 | 39.8 | 0.01419 | 1.3 | https://earthengine.google.org/#timelapse/v=6.94976,-71.98327,11.4,latLng |
| V9 | 7.03724 | -72.04633 | 1 | 13.3 | 677 | 19.6 | 0.00777 | 1.2 | https://earthengine.google.org/#timelapse/v=7.03724,-72.04633,11.4,latLng |
| V10 | 7.07250 | -72.00890 | 2 | 15.5 | 89 | 174.2 | 0.01923 | 1.0 | https://earthengine.google.org/#timelapse/v=7.0725,-72.0089,11.4,latLng |
| V11 | 7.17030 | -71.34500 | 3 | 82.4 | 110 | 749.1 | 0.00058 | 16.0 | https://earthengine.google.org/#timelapse/v=7.1703,-71.345,11.4,latLng |
| V12_1 | 7.49590 | -72.03500 | 1 | 4.8 | 150 | 32.0 | 0.01950 | 0.8 | https://earthengine.google.org/#timelapse/v=7.4959,-72.035,11.4,latLng |
| V12_2 | 7.49590 | -72.03500 | 1 | 4.8 | 150 | 32.0 | 0.01950 | 1.4 | https://earthengine.google.org/#timelapse/v=7.4959,-72.035,11.4,latLng |
| V12_3 | 7.49590 | -72.03500 | 1 | 4.8 | 150 | 32.0 | 0.01950 | 1.3 | https://earthengine.google.org/#timelapse/v=7.4959,-72.035,11.4,latLng |
| V13 | 7.55960 | -72.07990 | 1 | 1.8 | 670 | 2.7 | 0.07698 | 0.7 | https://earthengine.google.org/#timelapse/v=7.5596,-72.0799,11.4,latLng |
| V14 | 8.41460 | -69.98310 | 3 | 69.7 | 90 | 774.4 | 0.00146 | 25.4 | https://earthengine.google.org/#timelapse/v=8.4146,-69.9831,11.4,latLng |
| V15 | 7.23583 | -71.94778 | 1 | 16.9 | 426 | 39.7 | 0.02400 | 0.5 | https://earthengine.google.org/#timelapse/v=7.23583,-71.94778,11.4,latLng |
| B1 | -17.08150 | -62.97950 | 3 | 249.0 | 400 | 622.5 | 0.00049 | 38.9 | https://earthengine.google.org/#timelapse/v=-17.0815,-62.9795,11.4,latLng |
| B2 | -17.42410 | -64.13510 | 3 | 4.1 | 60 | 68.3 | 0.01500 | 0.4 | https://earthengine.google.org/#timelapse/v=-17.4241,-64.1351,11.4,latLng |
| B3 | -17.37140 | -64.21650 | 1 | 7.3 | 530 | 13.8 | 0.04889 | 1.1 | https://earthengine.google.org/#timelapse/v=-17.3714,-64.2165,11.4,latLng |
| B4 | -17.21600 | -64.85500 | 1 | 8.6 | 888 | 9.7 | 0.01460 | 1.5 | https://earthengine.google.org/#timelapse/v=-17.216,-64.855,11.4,latLng |
| B6 | -16.97290 | -65.39850 | 1 | 1.3 | 1540 | 0.9 | 0.00366 | 1.0 | https://earthengine.google.org/#timelapse/v=-16.9729,-65.3985,11.4,latLng |

| | | | | | | | | | |
|---|---|---|---|---|---|---|---|---|---|
| B7 | -16.70480 | -65.65000 | 3 | 3.1 | 132 | 23.5 | 0.03263 | 0.9 | https://earthengine.google.org/#timelapse/v=-16.7048,-65.65,11.4,latLng |
| B8 | -16.61250 | -65.74380 | 2 | 2.5 | 113 | 22.1 | 0.00596 | 1.1 | https://earthengine.google.org/#timelapse/v=-16.6125,-65.7438,11.4,latLng |
| B9 | -16.32250 | -65.83840 | 3 | 60.8 | 159 | 382.4 | 0.00103 | 3.3 | https://earthengine.google.org/#timelapse/v=-16.3225,-65.8384,11.4,latLng |
| B10 | -16.27280 | -65.20380 | 3 | 82.9 | 164 | 505.5 | 0.00068 | 13.9 | https://earthengine.google.org/#timelapse/v=-16.2728,-65.2038,11.4,latLng |
| B11 | -16.06620 | -66.15720 | 3 | 19.1 | 38 | 502.6 | 0.00212 | 1.5 | https://earthengine.google.org/#timelapse/v=-16.0662,-66.1572,11.4,latLng |
| B12 | -15.87900 | -65.85900 | 3 | 49.5 | 98 | 505.1 | 0.00075 | 12.8 | https://earthengine.google.org/#timelapse/v=-15.879,-65.859,11.4,latLng |
| B13 | -15.86680 | -66.36140 | 3 | 10.4 | 112 | 92.9 | 0.00306 | 0.8 | https://earthengine.google.org/#timelapse/v=-15.8668,-66.3614,11.4,latLng |
| B14 | -14.67700 | -66.53080 | 3 | 134.0 | 110 | 1218.2 | 0.00021 | 53.7 | https://earthengine.google.org/#timelapse/v=-14.677,-66.5308,11.4,latLng |
| B15 | -23.07690 | -64.31870 | 1 | 19.3 | 355 | 54.4 | 0.00295 | 1.0 | https://earthengine.google.org/#timelapse/v=-23.0769,-64.3187,11.4,latLng |
| H1 | 27.41970 | 94.24130 | 1 | 14.9 | 369 | 40.4 | 0.00059 | 0.9 | https://earthengine.google.org/#timelapse/v=27.4197,94.2413,11.4,latLng |
| H2 | 27.67860 | 94.85750 | 1 | 9.1 | 176.5 | 51.6 | 0.00081 | 2.0 | https://earthengine.google.org/#timelapse/v=27.6786,94.8575,11.4,latLng |
| H3 | 26.91380 | 86.04600 | 1 | 2.3 | 121 | 19.0 | 0.00682 | 1.2 | https://earthengine.google.org/#timelapse/v=26.9138,86.046,11.4,latLng |
| H4 | 29.38049 | 78.89051 | 1 | 7.8 | 70 | 111.4 | 0.00441 | 0.8 | https://earthengine.google.org/#timelapse/v=29.380488,78.890506,11.4,latLng |
| H5 | 26.78360 | 90.95660 | 1 | 0.4 | 1500 | 0.3 | 0.00165 | 0.8 | https://earthengine.google.org/#timelapse/v=26.7836,90.9566,11.4,latLng |
| H6 | 27.49620 | 96.21850 | 1 | 17.1 | 980 | 17.4 | 0.00383 | 1.4 | https://earthengine.google.org/#timelapse/v=27.4962,96.2185,11.4,latLng |
| G1 | -5.72600 | 145.52700 | 2 | 59.5 | 154 | 386.4 | 0.00187 | 2.2 | https://earthengine.google.org/#timelapse/v=-5.726,145.527,11.4,latLng |
| G2 | -5.15780 | 144.78870 | 2 | 8.1 | 37.6 | 215.4 | 0.00255 | 9.5 | https://earthengine.google.org/#timelapse/v=-5.1578,144.7887,11.4,latLng |
| G3 | -9.64820 | 148.73160 | 2 | 3.1 | 111 | 27.9 | 0.00375 | 1.2 | https://earthengine.google.org/#timelapse/v=-9.6482,148.7316,11.4,latLng |
| G4 | -9.62060 | 148.65000 | 2 | 3.2 | 24 | 132.9 | 0.00494 | 1.6 | https://earthengine.google.org/#timelapse/v=-9.6206,148.65,11.4,latLng |
| G5 | -4.15120 | 135.24400 | 2 | 8.6 | 360 | 23.9 | 0.00452 | 4.9 | https://earthengine.google.org/#timelapse/v=-4.1512,135.244,11.4,latLng |
| G6 | -3.96930 | 135.21810 | 1 | 4.1 | 330 | 12.6 | 0.01113 | 0.7 | https://earthengine.google.org/#timelapse/v=-3.9693,135.2181,11.4,latLng |
| G7 | -4.00730 | 135.25140 | 1 | 0.1 | 87.4 | 1.1 | 0.01507 | 1.5 | https://earthengine.google.org/#timelapse/v=-4.0073,135.2514,11.4,latLng |
| G8 | -3.96860 | 135.07490 | 3 | 22.2 | 82 | 270.2 | 0.00334 | 13.1 | https://earthengine.google.org/#timelapse/v=-3.9686,135.0749,11.4,latLng |
| G9 | -3.04910 | 136.37110 | 1 | 6.3 | 124 | 51.1 | 0.00501 | 1.5 | https://earthengine.google.org/#timelapse/v=-3.0491,136.3711,11.4,latLng |
| G10 | -3.35350 | 138.75940 | 2 | 6.3 | 59 | 106.1 | 0.01534 | 0.5 | https://earthengine.google.org/#timelapse/v=-3.3535,138.7594,11.4,latLng |
| G11 | -6.57620 | 146.69910 | 1 | 9.7 | 219 | 44.3 | 0.00487 | 0.7 | https://earthengine.google.org/#timelapse/v=-6.5762,146.6991,11.4,latLng |

| | | | | | | | | | |
|---|---|---|---|---|---|---|---|---|---|
| G12 | -6.66240 | 147.05690 | 2 | 4.0 | 148 | 27.0 | 0.01329 | 2.5 | https://earthengine.google.org/#timelapse/v=-6.6624,147.0569,11.4,latLng |
| G13 | -6.69530 | 147.21110 | 1 | 4.0 | 231 | 17.3 | 0.01132 | 1.7 | https://earthengine.google.org/#timelapse/v=-6.6953,147.2111,11.4,latLng |
| G14 | -6.69310 | 147.33000 | 1 | 2.3 | 405 | 5.7 | 0.01616 | 0.7 | https://earthengine.google.org/#timelapse/v=-6.6931,147.33,11.4,latLng |
| G15 | -7.57740 | 147.23590 | 1 | 0.1 | 168 | 0.6 | 0.00469 | 0.7 | https://earthengine.google.org/#timelapse/v=-7.5774,147.2359,11.4,latLng |
| G16 | -7.67100 | 147.42650 | 2 | 3.1 | 182 | 17.0 | 0.00226 | 1.0 | https://earthengine.google.org/#timelapse/v=-7.671,147.4265,11.4,latLng |
| G17 | -9.07900 | 149.04070 | 1 | 5.7 | 215 | 26.5 | 0.00410 | 1.4 | https://earthengine.google.org/#timelapse/v=-9.079,149.0407,11.4,latLng |
| G18 | -9.62930 | 149.30820 | 1 | 6.5 | 220 | 29.5 | 0.00947 | 0.9 | https://earthengine.google.org/#timelapse/v=-9.6293,149.3082,11.4,latLng |
| G19 | -4.97120 | 139.91190 | 1 | 7.4 | 137 | 54.0 | 0.00502 | 0.9 | https://earthengine.google.org/#timelapse/v=-4.9712,139.9119,11.4,latLng |
| G20 | -5.06240 | 139.72730 | 3 | 22.9 | 96 | 238.5 | 0.00330 | 1.7 | https://earthengine.google.org/#timelapse/v=-5.0624,139.7273,11.4,latLng |
| G21 | -4.72310 | 139.30090 | 1 | 7.0 | 752 | 9.3 | 0.00499 | 0.7 | https://earthengine.google.org/#timelapse/v=-4.7231,139.3009,11.4,latLng |
| G22 | -4.68700 | 138.78890 | 2 | 6.7 | 164 | 40.9 | 0.00138 | 1.6 | https://earthengine.google.org/#timelapse/v=-4.687,138.7889,11.4,latLng |
| G23 | -4.36630 | 136.84750 | 1 | 9.7 | 290 | 33.4 | 0.00509 | 0.6 | https://earthengine.google.org/#timelapse/v=-4.3663,136.8475,11.4,latLng |
| G24 | -4.34880 | 136.79780 | 1 | 6.4 | 360 | 17.8 | 0.00758 | 1.2 | https://earthengine.google.org/#timelapse/v=-4.3488,136.7978,11.4,latLng |
| G25_1 | -0.76726 | 133.62207 | 2 | 8.9 | 110 | 81.0 | 0.01113 | 1.0 | https://earthengine.google.org/#timelapse/v=-0.767259,133.622071,11.4,latLng |
| G25_2 | -0.80550 | 133.62000 | 1 | 5.4 | 310 | 17.4 | 0.01130 | 0.7 | https://earthengine.google.org/#timelapse/v=-0.8055,133.62,11.4,latLng |
| G26 | -3.31720 | 128.68310 | 3 | 1.5 | 205 | 7.3 | 0.00364 | 0.6 | https://earthengine.google.org/#timelapse/v=-3.3172,128.6831,11.4,latLng |

Supplementary Table 1. **Avulsion Style data**. Type refers to channel morphology as 1 – multithreaded, 2 – transitional, and 3 – single threaded. Normalized distance is the river distance (mountain front to avulsion site) divided by channel width.

| Landsat 5 | Band 1 | Band 2 | Band 3 | Band 4 | Band 5 | Band 7 |
|---|---|---|---|---|---|---|
| | 0.2909 | 0.2493 | 0.4806 | 0.5568 | 0.4438 | 0.1706 |
| | -0.2728 | -0.2174 | -0.5508 | 0.7221 | 0.0733 | -0.1648 |
| | 0.1446 | 0.1761 | 0.3322 | 0.3396 | -0.621 | -0.4186 |
| | 0.8461 | -0.0731 | -0.464 | -0.0032 | -0.0492 | 0.0119 |
| | 0.0549 | -0.0232 | 0.0339 | -0.1937 | 0.4162 | -0.7823 |
| | 0.1186 | -0.8069 | 0.4094 | 0.0571 | -0.0228 | 0.022 |
| | | | | | | |
| Landsat 7 | Band 1 | Band 2 | Band 3 | Band 4 | Band 5 | Band 7 |
| | 0.3561 | 0.3972 | 0.3904 | 0.6966 | 0.2286 | 0.1596 |
| | -0.3344 | -0.3544 | -0.4556 | 0.6966 | -0.0242 | -0.263 |
| | 0.2626 | 0.2141 | 0.0926 | 0.0656 | -0.7629 | -0.5388 |
| | 0.0805 | -0.0498 | 0.195 | -0.1327 | 0.5752 | -0.7775 |
| | -0.7252 | -0.0202 | 0.6683 | 0.0631 | -0.1494 | -0.0274 |
| | 0.4 | -0.8172 | 0.3832 | 0.0602 | -0.1095 | 0.0985 |
| | | | | | | |
| Landsat 8 | Band 1 | Band 2 | Band 3 | Band 4 | Band 5 | Band 7 |
| | 0.3029 | 0.2786 | 0.4733 | 0.5599 | 0.508 | 0.1872 |
| | -0.2941 | -0.243 | -0.5424 | 0.7276 | 0.0713 | -0.1608 |
| | 0.1511 | 0.1973 | 0.3283 | 0.3407 | -0.7117 | -0.4559 |
| | -0.8239 | 0.0849 | 0.4396 | -0.058 | 0.2013 | -0.2773 |
| | -0.3294 | 0.0557 | 0.1056 | 0.1855 | -0.4349 | 0.8085 |
| | 0.1079 | -0.9023 | 0.4119 | 0.0575 | -0.0259 | 0.0252 |

Supplementary Table 2. **Tasseled-cap coefficients**. Different sets of coefficients were derived for different landsat missions: Landsat 4-5 (ref. 1). Landsat 7 (ref. 2), and Landsat 8 (ref. 3). Coefficients are used to process band data to compress the multiple Landsat bands into three physically meaningful bands, including brightness, greenness, and wetness.

## Supplementary Figures

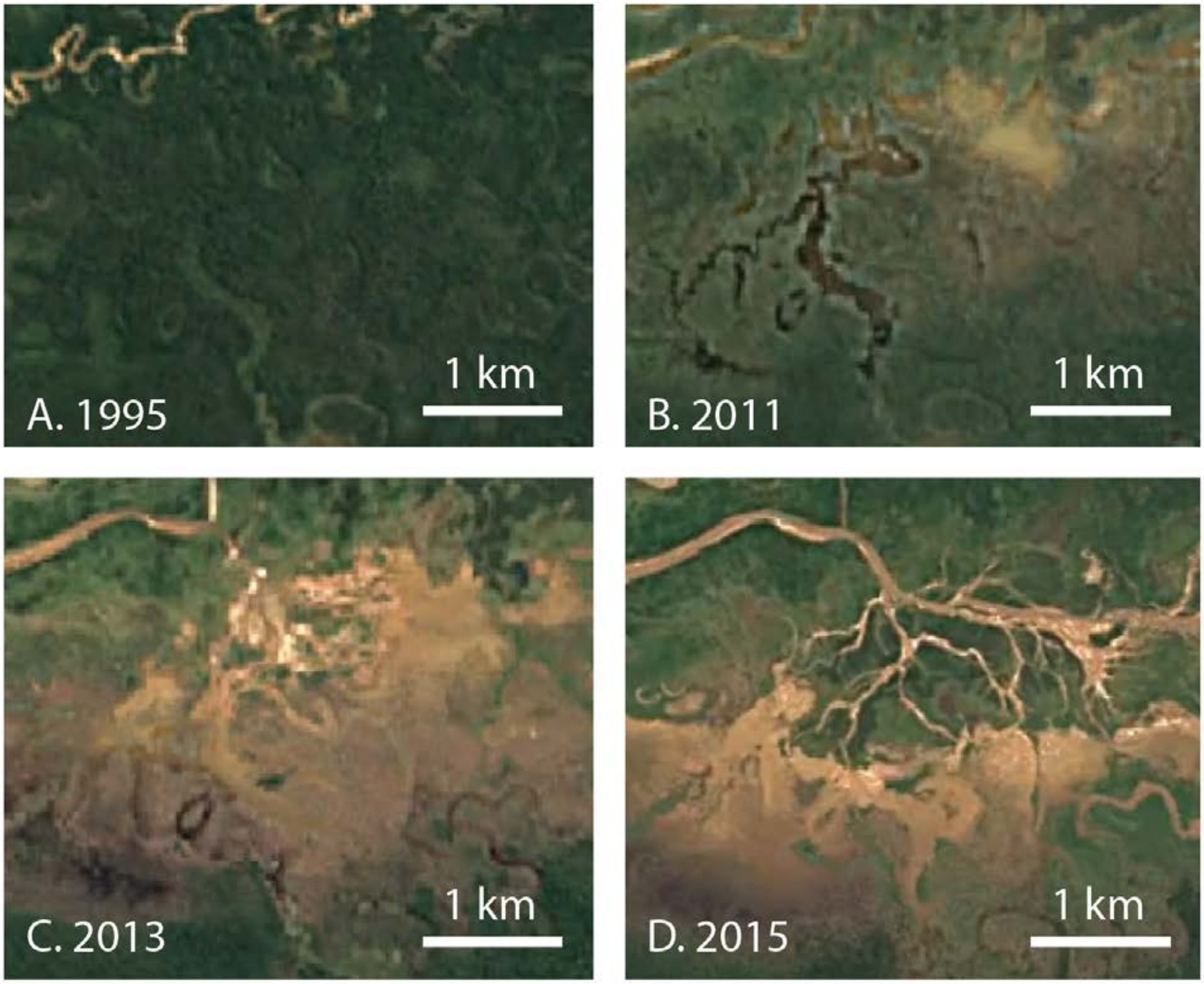


Supplementary Figure 1. **Progression of land surface changes during progradational event (sample B12, Bolivia)**. Changes in color correspond to shifts in the land surface spectra. A. undisturbed forest and parent channel (1995). B. Initial diversion of flow from parent channel results in flooding, in this case without a crevasse splay (2011). C. Incipient distributary network and expanding flooding area with vegetation removal (2013). D. Further development of distributary network and strengthening of a single distributary channel (2015). Annual composite images from www.earthengine.google.com/timelapse.

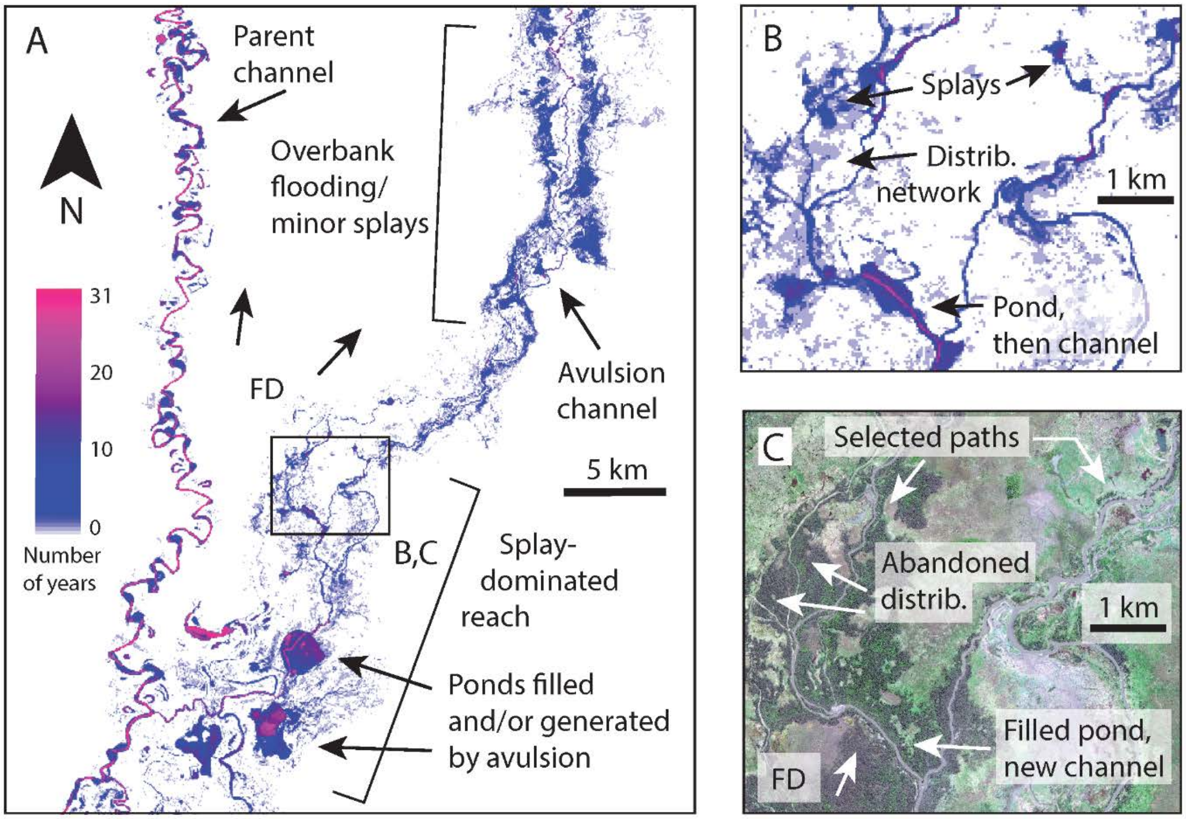


**Supplementary Figure 2. Qualitative validation of results from avulsion sample B11, Bolivia**. A. Avulsion fingerprint, where positively identified avulsion pixels from each annual composite are summed, constituting the "number of years", pink representing more years of activity, blue, fewer. Flow direction (FD) is to the north for the parent channel and to northeast for the avulsion channel. Two distinct reaches are visible, with the first dominated by splays and pond generation and filling, followed downstream by annexation accompanied by proximal overbank flooding and minor splays. B. Subset of avulsion fingerprint within splay dominated reach showing morphological details including splays, distributary network, and ponding. C. High-resolution image of the same subset area, July 8, 2016. Selected and abandoned channel flow paths are annotated. Note areas covered by splays are later colonized by dense vegetation (dark green). World View 1 RGB image from Digital Globe.

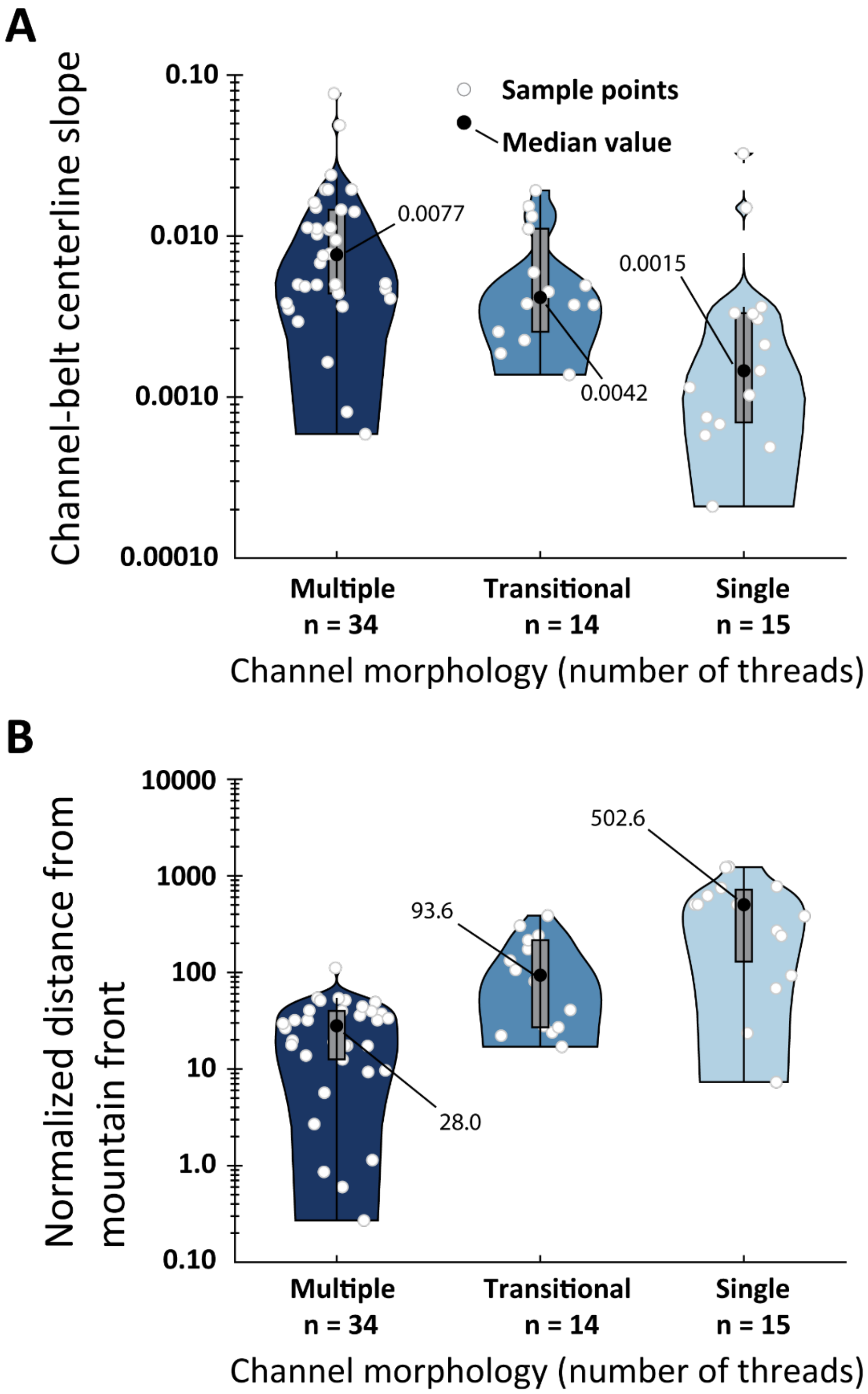


**Supplementary Figure 3. Channel morphology is related to slope and normalized distance.** Avulsion sites are used as points of reference for the following comparisons of channel characteristics: A. Channel morphologies exhibit unique distributions and medians for slope

along the channel belt centerline. Multithread channels have the highest median value at 0.0077, and show the most skew towards higher slope values. Events in transitional channels have median $S_R$ value of 0.0042, with the least skew. Single-thread channels have the lowest median value at 0.0015. B. Channel morphologies exhibit even more discrete distributions and medians for normalized distance (length/width) from the mountain front. Multithread channels have the lowest median distance value of 28.0, transitional channels again have a value of 93.6, and single-thread channels have the highest median distance value of 502.6. These trends follow the simple generalization that rivers tend to exhibit multithread morphology in more proximal locations with higher slopes, and single-thread morphology in more distal locations with lower slopes. Numbers refer to median values of the distributions, boxes show the 2$^{nd}$ to 3$^{rd}$ quartile ranges, and lines extending from boxes represent the 1$^{st}$ to 2$^{nd}$ and 3$^{rd}$ to 4$^{th}$ quartile ranges.

## References


1 Kauth, R. J. & Thomas, G. in *LARS symposia*. 159.

2 Crist, E. P. & Cicone, R. C. A physically-based transformation of Thematic Mapper data---The TM Tasseled Cap. *IEEE Transactions on Geoscience Remote Sensing*, 256-263 (1984).

3 Baig, M. H. A., Zhang, L., Shuai, T. & Tong, Q. Derivation of a tasselled cap transformation based on Landsat 8 at-satellite reflectance. *Remote Sensing Letters* **5**, 423-431 (2014).
