# Supplementary material for: Downstream changes in river avulsion style are related to channel morphology
Source: Nat Commun. 2020 Apr 30;11:2116. doi: 10.1038/s41467-020-15859-9 (PMC7192919; doi:10.1038/s41467-020-15859-9)
Supplement: Supplementary file 2 — Description of Additional Supplementary Files [file 41467_2020_15859_MOESM2_ESM.pdf]

Description of Additional Supplementary Files

File Name: Supplementary Data 1
Description: Avulsion locations and parameters measured in this study. See Notes tab in the dataset for detailed explanation of each data column.
